# Analysis of the predictive value of the Geriatric Nutritional Risk Index for osteoporosis in elderly patients with T2DM: a single-center retrospective study

**DOI:** 10.1186/s13018-023-04237-y

**Published:** 2023-10-07

**Authors:** Silu Sun, Simin Tao, Xiaoyan Xi, Tao Jiang, Qian Zhu, Yan Zhou, Hui Li

**Affiliations:** 1 School of Nursing, Chengdu Medical College, Tianhui Road, Chengdu, 610083 China; 2 Department of Orthopedics, The First Affiliated Hospital of Chengdu Medical College, 278 Baoguang Avenue, Middle Section, Chengdu, 610599 China

**Keywords:** Geriatric Nutritional Risk Index, Bone mineral density, Osteoporosis, T2DM, Nutritional assessment

## Abstract

**Background:**

Malnutrition is recognized as a risk factor for osteoporosis and T2DM. Previous studies have demonstrated the relationship between nutritional assessment tools and BMD. However, few studies have compared the effects of three nutritional risk assessment tools (GNRI, CONUT, and PNI). This study aimed to investigate the correlation between three nutritional assessment tools and BMD and to compare their validity in predicting osteoporosis in type 2 diabetes mellitus in the elderly.

**Methods:**

This retrospective study collected clinical data from 525 elderly patients with type 2 diabetes mellitus and categorized the patients into osteoporotic and non-osteoporotic groups. The correlation between the three nutritional assessment tools and BMD was analyzed using Spearman partial correlation. Binary logistics regression was used to analyze the relationship between GNRI and osteoporosis. ROC curves were used to compare the validity of GNRI, PNI, and CONUT in predicting osteoporosis.

**Results:**

Spearman’s partial correlation showed a positive correlation between femoral neck BMD and lumbar spine BMD, but no correlation was observed between total hip BMD and GNRI. Logistic regression analyses showed no association between PNI, CONUT scores, and the development of osteoporosis. After adjusting for age, sex, smoking, alcohol consumption, BMI, ALB, Cr, UA, FBG, TG, and HDL, the correlation between GNRI and osteoporosis remained. ROC curve analysis showed that GNRI in combination with age and albumin had better predictive ability for osteoporosis than PNI and CONUT.

**Conclusion:**

GNRI was an independent protective factor against osteoporosis in elderly patients with T2DM, and the predictive ability of GNRI for osteoporosis in elderly patients with T2DM was better than that of PNI and CONUT scores.

## Introduction

Type 2 diabetes mellitus (T2DM) and osteoporosis (OP) are two major metabolic diseases commonly seen in elderly patients. As we all know, osteoporosis is a metabolic disease that is affected by age, hormones, and other factors leading to decreased bone mass and impaired bone microstructure, resulting in increased bone fragility and even fractures. Osteoporosis and the ensuing fragility fractures are a huge economic burden for many countries [1]. Previous studies have identified many risk factors for osteoporosis and therapeutic drugs have been developed, such as bisphosphonates, teriparatide, and linagliptin [2–4]. In fact, only a small proportion of patients have been treated [5]. A cross-sectional study in mainland China reported that T2DM affects more than 74.22 million people in China [6]. A persistent hyperglycemic state is positively associated with osteoporosis risk [7]. Patients with long disease duration may be at higher risk of falls and fractures due to factors such as peripheral neuropathy, visual impairment, and cardiovascular disease [8]. Therefore, the management of osteoporosis and T2DM is of increasing interest. Poor nutritional status is an important risk factor for the development of T2DM and osteoporosis. A study by SHANGGUAN et al. illustrated that patients with osteoporosis in the presence of nutritional deficiencies have a higher risk of all-cause mortality [9], and diabetic patients in the presence of nutritional deficiencies have a poorer quality of life than nondiabetic patients [10].

The Geriatric Nutritional Risk Index (GNRI) is a tool developed by Bouillanne and used to identify the risk of nutrition-related complications in elderly hospitalized patients [11], which is calculated from height, weight, and albumin. The tool is less limited by physical status and excludes the interference of subjective factors. Similar to the GNRI, the Control Nutrition Status (CONUT) score and the Prognostic Nutrition Index (PNI) are objective nutritional assessment tools. These tools are widely used in the field of cardiovascular diseases, oncology, and osteoporotic fractures [12–14]. Similarly, several studies have compared the validity of the three tools [14, 15]. However, few studies have reported the validity of the three nutritional risk assessment tools in predicting osteoporosis in the elderly with T2DM. Therefore, this study aimed to investigate the correlation between the three nutritional assessment tools and BMD at all sites (femoral neck, lumbar spine, and total hip) and compare their validity in predicting osteoporosis in elderly patients with T2DM.

## Materials and methods

### Study patients

This was a single-center retrospective study on 525 patients with T2DM who were treated at the First Affiliated Hospital of Chengdu Medical College from August 2016 to July 2023. The inclusion criteria were as follows: patients aged 60 years or older; diagnosed with T2DM; received dual-energy X-ray absorptiometry to measure bone density (DXA), biochemical indexes, and nutritional assessment (GNRI, PNI, CONUT). The exclusion criteria were as follows: incomplete medical record information; end-stage disease, malignancy, immune system disease, severe liver insufficiency, chronic renal failure, moderate to severe anemia; thyroid or parathyroid dysfunction; individuals who have received treatment for osteoporosis; individuals who had taken thiazolidinedione, glucocorticoids, thyroid hormones, calcium and other drugs affecting bone metabolism in the last 6 months. The Chengdu Medical College Ethics Committee approved the study and complied with the Declaration of Helsinki (CMCEC–2022N0.40). Patient informed consent was not required due to the retrospective design of this study.

### Clinical data

The hospital’s electronic medical record system collected all clinical record data. Demographic information included age, gender, blood pressure, smoking, alcohol consumption, height, weight, and BMI. Height was measured without shoes and weight was measured with light clothing. Body mass index was calculated for each patient by dividing the measured weight by the square of the height (kg/m^2^). Patients were asked to refrain from strenuous exercise or emotional stress for half an hour before blood pressure measurements, to empty their bladders, and to rest properly for at least five minutes. Smoking was defined as continuous or cumulative smoking for six months or more, and drinking was defined as drinking alcohol at least once a week for six months or more [16]. Patients were fasted and dehydrated after 10 pm on the day of admission, and fasting venous blood was collected at 6 am on the next day. Laboratory measurements included serum total protein (TP), serum albumin (ALB), blood creatinine (Cr), blood uric acid (UA), serum calcium, serum phosphorus, fasting blood glucose (FBG), triglycerides(TG), total cholesterol(TC), high-density lipoprotein(HDL), low-density lipoprotein(LDL), total lymphocyte count(TLC).

### Bone mineral density

All patients underwent dual-energy X-ray absorptiometry (enCORE, General Electric Company, version 15) to determine the BMD (g/m^2^) of the femoral neck, lumbar spine (L1–L4), and left total hip. T-score > 2.5 standard deviations at any site were diagnosed as osteoporosis according to the 1994 WHO diagnostic criteria for osteoporosis [17].

### Nutritional assessment tools

The GNRI was calculated from height, weight, and serum albumin, which is used to assess the patient's nutritional status in the pathological state [18]. GNRI = [1.489 × albumin (g/L)] + [41.7 × (actual weight/ideal body weight)], ideal body weight(male) = height (cm) − 100 − [height (cm) − 150]/4], ideal body weight(female) = height (cm) − 100 − [height (cm) − 150]/2.5]. When the actual body weight exceeds the ideal body weight, the actual body weight/ideal body weight is set to 1, otherwise it is recorded as the actual value [19]. The PNI was calculated based on serum albumin and total lymphocyte count [20]. PNI = serum albumin (g/L) + 5 × total lymphocyte count (10^9^/L). The CONUT score was calculated based on serum albumin, total cholesterol, and total lymphocyte count, serum albumin (g/L) 35 or greater (0 points), 30–34.9 (2 points), 25–29.9 (4 points), and 25 or below (6 points); total cholesterol (mg/dL)180 or greater (0 points), 140–179 (1 point), 100 to 139 (2 points), and 100 or below (3 points); total lymphocyte count (10^9^/L) 1.6 or greater (0 points), 1.2–1.59 (1 point), 0.8–1.19 (2 points), and 0.8 or below (3 points) [21]. The criteria for malnutrition are as follows: GNRI < 98, CONUT score > 2, PNI < 45.

### Statistical analysis

Continuous variables were expressed as mean ± standard and median (25th percentile, 75th percentile). The difference in a quantitative variable between the two groups was investigated using the unpaired t test or Mann–Whitney’s U test. Categorical data were expressed as frequencies (%), and the difference between the two groups was determined using the χ^2^ test. Spearman's partial correlation was used to determine the correlation between GNRI, PNI, CONUT score, and bone mineral density. Binary logistic regression analysis was used to determine the relationship between GNRI and osteoporosis. Variable with a statistically significant difference between the groups (*P* < 0.005) and/or potential confounding factors known to have an effect on bone health (*P* < 0.20 in the univariate analyses) were considered for inclusion in the final model. Variance inflation factors (VIF) were used for covariance diagnostics and to exclude variables with VIF above 2. Based on the above criteria, we included the following covariates: age, sex, smoking, drinking, BMI, ALB, Cr, UA, FBG, TG, HDL, and GNRI. In males and females, ROC curves were applied to compare the predictive value of the three nutritional assessment tools for osteoporosis, and the area under the curve was calculated, respectively. All statistical analysis was carried out using IBM SPSS Statistics version 26.0 (IBM Corporation; Armonk, NY, USA) and MedCalc 20.03. A *P* value < 0.05 was considered statistically significant.

## Results

**Fig. 1:**
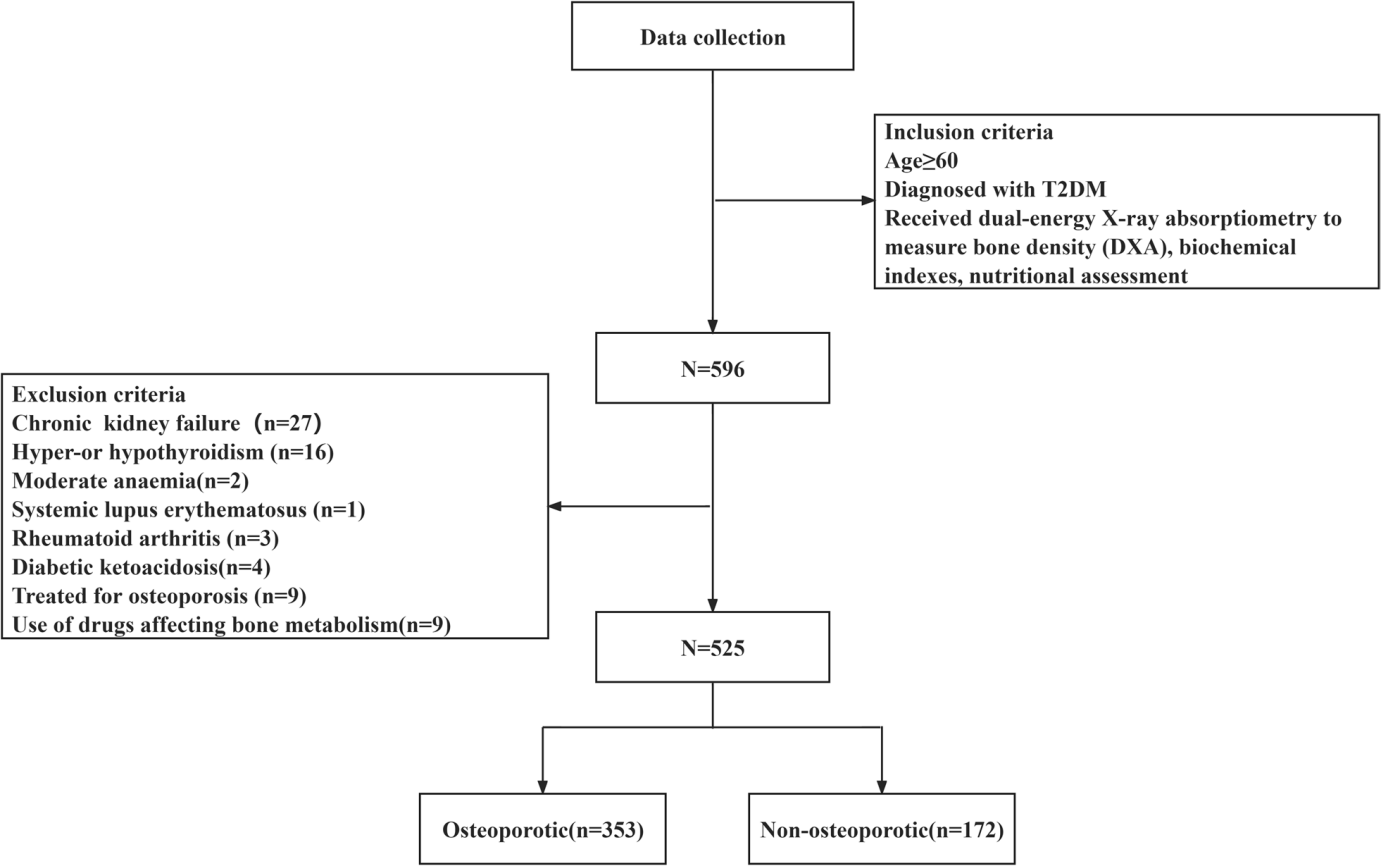
Flowchart of participant enrollment

The participants were selected as shown in Fig. 1, the group was divided into osteoporotic and non-osteoporotic groups. A total of 525 patients met the inclusion criteria for this retrospective study, 71 patients were excluded because of the exclusion criteria, and the basic characteristics of the patients are shown in Table 1. A total of 353 osteoporotic and 172 non-osteoporotic patients, 80.8% of whom were female. 8.7% had a history of smoking and 7.9% of drinking. The mean age of 71.6 ± 6.9 years and the BMI of 23.56 ± 3.42 (kg/m^2^) are observed. The mean GNRI was 100.68 ± 7.64, the mean CONUT was 2.27 ± 1.76, and the mean PNI was 17.71 ± 5.86. Femoral neck BMD, lumbar spine BMD, and total hip BMD were 0.73 ± 0.14, 0.88 ± 0.16, and 0.81 ± 0.18, respectively. Osteoporotic patients were older (72.1 ± 7.0 vs 70.5 ± 6.6, *P* = 0.013) and had lower femoral neck BMD(0.70 ± 0.13 vs 0.79 ± 0.14, *P* < 0.001), lumbar spine BMD (0.83 ± 0.14 vs 0.98 ± 0.15, *P* < 0.001), and total hip BMD(0.78 ± 0.18 vs 0.87 ± 0.16, *P* < 0.001) compared to non-osteoporotic patients. Except for GNRI (100.22 ± 7.67 vs 101.62 ± 7.53, *P* = 0.049), the differences in the rest of the nutritional indicators were not statistically significant.

**Table 1:** Patient characteristic

Variables	All (*n* = 525)	Osteoporotic (*n* = 353)	Non-osteoporotic (*n* = 172)	*P* value
Age (year)	71.6 ± 6.9	72.1 ± 7.0	70.5 ± 6.6	**0.013**
Sex, female	424 (80.8)	306 (86.7)	118 (68.6)	**< 0.001**
High blood pressure, *n* (*%*)	297 (56.6)	203 (57.5)	94 (54.7)	0.536
Smoking, *n* (*%*)	45 (8.7)	25 (7.1)	20 (12.0)	0.065
Drinking, *n* (*%*)	39 (7.9)	22 (6.3)	17 (10.3)	0.108
BMI (kg/m^2^)	23.56 ± 3.42	23.37 ± 3.46	23.96 ± 3.33	0.061
TP (g/L)	71.67 (66.65,76.1)	71.80 (66.75,76.40)	71.25 (66.00,75.78)	0.443
ALB (g/L)	40.9 (37.7,43.6)	41.00 (37.60,43.75)	40.75 (37.73,43.30)	0.552
Cr (μmol/L)	63.80 (51.85,79.55)	62.5 (51.85,80.05)	68.80 (53.60,84.28)	0.102
UA (μmol/L)	316.00 (255.00,388.50)	317.00 (257.50,388.00)	311.50 (251.81,390.00)	0.791
Serum calcium (mmol/L)	2.34 (2.25,2.48)	2.35 (2.25,2.50)	2.34 (2.24,2.47)	0.372
Serum phosphorus (mmol/L)	1.12 (0.96,1.39)	1.11 (0.96,1.39)	1.13 (0.96,1.39)	0.925
FBG (mmol/L)	8.47 (5.61,12.80)	8.38 (5.45,12.92)	8.66 (6.05,12.69)	0.598
TG (mmol/L)	1.55 (1.09,2.30)	1.57 (1.11,2.26)	1.50 (1.06,2.32)	0.586
TC (mmol/L)	4.40 (3.66,5.15)	4.43 (3.71,5.19)	4.29 (3.58,5.05)	0.238
HDL-C (mmol/L)	1.27 (1.06,1.53)	1.28 (1.08,1.55)	1.22 (1.03,1.51)	0.061
LDL-C (mmol/L)	2.49 (1.84,3.23)	2.52 (1.87,3.24)	2.43 (1.79,3.20)	0.635
Femoral neck BMD (g/cm^2^)	0.73 ± 0.14	0.70 ± 0.13	0.79 ± 0.14	**< 0.001**
Lumbar spine BMD (g/cm^2^)	0.88 ± 0.16	0.83 ± 0.14	0.98 ± 0.15	**< 0.001**
Total Hip BMD (g/cm^2^)	0.81 ± 0.18	0.78 ± 0.18	0.87 ± 0.16	**< 0.001**
GNRI	100.68 ± 7.64	100.22 ± 7.67	101.62 ± 7.53	**0.049**
CONUT	2.27 ± 1.76	2.24 ± 1.76	2.35 ± 1.76	0.465
PNI	47.71 ± 5.86	47.64 ± 5.74	47.85 ± 6.10	0.693

Bold value indicate significant *P*-values

*TP* Total serum protein, *BMI* Body mass index, *ALB* Serum albumin, *Cr* Creatinine, *UA* Hematuria, *FBG* Fasting blood glucose, *TG* Triglycerides, *TC* Total cholesterol, *HDL* High-density lipoprotein, *LDL* Low-density lipoprotein, *BMD* Bone mineral density, *GNRI* Geriatric Nutritional Risk Index, *CONUT* Controlling Nutritional Status, *PNI* Prognostic Nutrition Index

Spearman’s partial correlation showed a positive correlation between femoral neck BMD, lumbar spine BMD and GNRI after adjusting for age, but no correlation was observed between total hip BMD and GNRI (Table 2). BMI was positively correlated with BMD at all sites, with correlation coefficients of 0.136, 0.009, and 0.140 (*P* < 0.05); however, HDL was negatively correlated with correlation coefficients of − 0.125, − 0.128, and − 0.130 (*P* < 0.05).

**Table 2:** Spearman's partial correlation analysis of bone mineral density with nutritional assessment tools and biochemical indicators

Variables	Femoral neck BMD		Lumbar spine BMD		Total Hip BMD	
	Correlation coefficient, r	*P*	Correlation coefficient, r	*P*	Correlation coefficient, r	*P*
BMI	0.136	**0.002**	0.099	**0.024**	0.140	**0.002**
TP	0.104	**0.018**	0.023	0.603	0.085	**0.066**
ALB	0.070	0.111	0.043	0.331	0.032	0.468
Cr	− 0.034	0.443	0.049	0.264	− 0.034	0.432
UA	0.040	0.364	0.071	0.104	0.026	0.554
Serum calcium	− 0.099	**0.023**	− 0.091	**0.037**	− 0.026	0.559
Serum phosphorus	− 0.061	0.162	− 0.065	0.139	0.000	0.996
FBG	0.011	0.798	− 0.023	0.601	− 0.024	0.577
TG	− 0.104	**0.018**	− 0.072	0.099	− 0.070	0.107
TC	− 0.098	**0.024**	− 0.102	**0.019**	− 0.052	0.239
HDL-C	− 0.125	**0.004**	− 0.128	**0.003**	− 0.130	**0.003**
LDL-C	− 0.079	0.071	− 0.080	0.067	− 0.043	0.330
GNRI	0.103	**0.019**	0.088	**0.045**	0.077	0.080
CONUT	− 0.008	0.861	− 0.037	0.402	− 0.022	0.608
PNI	0.038	0.390	0.046	0.297	0.014	0.750

Bold value indicate significant *P*-values

*BMI* Body mass index, *TP* Total serum protein, *ALB* Serum albumin, *Cr* Creatinine, *UA* Hematuria, *FBG* Fasting blood glucose, *TG* Triglycerides, *TC* Total cholesterol, *HDL* High-density lipoprotein, *LDL* Low-density lipoprotein, *BMD* Bone mineral density, *GNRI* Geriatric Nutritional Risk Index, *CONUT* Controlling Nutritional Status, *PNI* Prognostic Nutrition Index

Binary logistic regression was used to analyze the relationship between nutritional assessment tools and osteoporosis. The results showed no relationship between CONUT, PNI, and osteoporosis. After adjusting for age, sex, smoking, drinking, BMI, ALB, Cr, UA, FBG, TG, HDL, and GNRI remained associated with osteoporosis (Table 3).

**Table 3:** Binary logistics regression analysis of the relationship between nutritional assessment tools and osteoporosis

	Model 1			Model 2			Model 3		
	OR	95% CI	*P* value	OR	95% CI	*P* value	OR	95% CI	*P* value
GNRI	0.971	0.946–1.000	**0.050**	0.971	0.971–0.946	**0.027**	0.948	0.948–0.913	**0.005**
CONUT	0.962	0.869–1.006	0.465	0.992	0.890–1.105	0.884	0.988	0.873–1.117	0.841
PNI	0.994	0.963–1.025	0.693	0.986	0.954–1.019	0.402	0.956	0.895–1.021	0.178

Bold value indicate significant *P*-values

Model 1: unadjusted model

Model 2: Model 1 + adjusted for sex, smoking, and drinking

Model 3: Model 2 + adjusted for age, BMI, ALB, Cr, UA, FBG, TG, HDL

*GNRI* Geriatric Nutritional Risk Index, *CONUT* Controlling Nutritional Status, *PNI* Prognostic Nutrition Index

The results of the ROC curve analysis demonstrated the ability of the three nutritional assessment tools to predict osteoporosis when combined with age and albumin, respectively. The GNRI was superior to the CONUT and PNI in predicting osteoporosis in older adults with type 2 diabetes mellitus. In males, the AUC for the prediction of osteoporosis was 0.688 with a sensitivity of 0.758 and a specificity of 0.588. In females, the AUC was 0.644, sensitivity was 0.561, and specificity was 0.670 (Table 4).

**Table 4:** Comparing the ability of nutritional assessment tools to predict osteoporosis

	AUC		95% CI		Sensitivity		Specificity	
	Male	Female	Male	Female	Male	Female	Male	Female
GNRI combined age and albumin	0.688	0.644	0.588–0.777	0.596–0.690	0.758	0.561	0.588	0.670
CONUT combined age and albumin	0.576	0.635	0.473–0.674	0.587–0.681	0.939	0.462	0.221	0.759
PNI combined age and albumin	0.576	0.640	0.474–0.674	0.592–0.685	0.303	0.516	0.868	0.714

*ROC* Receiver operating characteristic, *AUC* area under the curve, *CONUT* Controlling Nutritional Status, *GNRI* Geriatric Nutritional Risk Index, *PNI* prognostic nutritional index

The results of the ROC curve analysis demonstrated the ability of the three nutritional assessment tools to predict osteoporosis when combined with age and albumin, respectively. The GNRI was superior to the CONUT and PNI in predicting osteoporosis in older adults with type 2 diabetes mellitus. In males, the AUC for the prediction of osteoporosis was 0.688 with a sensitivity of 0.758 and a specificity of 0.588. In females, the AUC was 0.644, sensitivity was 0.561 and specificity was 0.670 (Table 4).

## Discussion

In this study, the mean age of the sample was 71.6 ± 6.9 years, 80.8% were female, 56.6% had hypertension, and the prevalence of osteoporosis was 67.2%. We observed that femoral neck BMD (*P* < 0.001), lumbar spine BMD (*P* < 0.001), and total hip BMD (*P* < 0.001) were higher in non-osteoporotic patients than in osteoporotic patients. The difference in GNRI (*P* = 0.049) scores between the osteoporotic and non-osteoporotic groups was statistically significant, and the difference in CONUT (*P* = 0.465) and PNI (*P* = 0.693) scores was not statistically significant.

We further analyzed the correlation of BMD with the three nutritional assessment tools and the potential association of BMD with each biochemical indicator. Since osteoporosis is an age-accelerating disease, we controlled for age as a potential confounder. Several previous studies have described the correlation between GNRI and osteoporosis. JI et al. reported that GNRI was positively correlated with femoral neck BMD, lumbar spine BMD, and total hip BMD [22] and that GNRI was a better predictor of osteoporosis in elderly patients with T2DM than albumin and BMI [23]. In contrast to previous studies, our study only observed a correlation between GNRI and femoral neck BMD and lumbar spine BMD, and GNRI did not correlate with total hip BMD. We considered that the reason for this result was influenced by the measurement position. According to Xu, when DXA is used to measure hip BMD, the patient's body is placed in a natural position and the BMD value is higher than that in the internal rotation position (the femoral stem is internally rotated by 15° to 25° together with the feet) [24]. So an incorrect measurement position may result in an inaccurate BMD.

Binary Logistic regression analysis showed a significant correlation between GNRI and osteoporosis after adjusting for age, gender, smoking, drinking, BMI, ALB, Cr, UA, TG, FBG, and HDL. Age is one of the important factors triggering osteoporosis. The balance between osteoblasts and osteoclasts is disrupted with increasing age, with osteoblasts becoming progressively less active and osteoclasts active. Typically, bone mass peaks before the age of 30 and then gradually declines. Previous studies have demonstrated a strong link between nutritional status and BMD [25]. We observed a positive correlation between BMI and BMD at all sites (*P* = 0.004, *P* = 0.003,* P* = 0.003, respectively). The same result was obtained with the study of Irene Zolfaroli et al. This result can be explained by the fact that an increased BMI leads to an increased mechanical load on the body, stimulating bone formation [26]. In addition, certain metabolic substances are involved in the process of bone metabolism. For example, tumor necrosis factor and interleukin 6 play a role in promoting osteoclast differentiation [27], but these factors are associated with abdominal obesity [28]. The positive effect of BMI on BMD is limited. The positive effect of increased BMI on BMD is limited to BMI < 35 kg/m^2^ or less, and there is no effect on the increase of BMD when BMI exceeds 35 kg/m^2^ [29]. A Mendelian randomization study demonstrated a negative effect of obesity on BMD [30]. Therefore, we believe that the damage caused by overweight/obesity far outweighs the positive effects on BMD. Past studies have confirmed that overweight/obesity is a risk factor for various cardiovascular [31, 32] and endocrine-metabolic diseases [31] as well as fractures [33].

Malnutrition is a risk factor for various complications and poor disease prognosis and is closely related to a patient's quality of life [34, 35]. Early assessment of nutritional status is important for diagnosing nutritional deficiencies and improving the prognosis of patients. The results of the ROC curve analysis showed that the GNRI combined with age and albumin had the best ability to predict osteoporosis compared with the PNI and CONUT scores. The GNRI, which is calculated by the combination of height, weight, and serum albumin [11], has received a lot of attention from researchers due to its ease of operation and less influence by subjective factors. The GNRI has received attention from researchers because it is easy to perform and less affected by subjective factors, and is useful for the prediction of prognosis and mortality in many diseases [36, 37].

In our study, HDL was negatively correlated with BMD at all sites (*P* = 0.002, *P* = 0.024,* P* = 0.002, respectively), and total cholesterol was negatively correlated with femoral neck BMD (*P* = 0.024) and lumbar spine BMD (*P* = 0.019). The results of studies on the relationship between serum lipids and BMD are currently controversial. Some studies have reported a negative correlation between HDL-C and BMD [38, 39]. Some studies have found a positive correlation between HDL-C and lumbar spine BMD and total hip BMD [40], and that HDL-C is a protective factor for OP in patients with T2DM [41]. However, some studies also reported no relationship [42]. We hypothesize that this discrepancy may be due to racial and gender differences in sample size.

It has been demonstrated that smoking and alcohol consumption affect bone metabolism and damage bone microarchitecture, although these two factors were not significant in this study. Tobacco contains high levels of tar and nicotine metabolites, and damage to bone microarchitecture is evident. Li et al. found that cadmium in smoke was shown to interfere with bone metabolism directly or indirectly in both animal and human experiments [43]. In contrast, smokers showed significantly higher levels of bone formation markers, such as osteocalcin and uncarboxylated osteocalcin, within 124 days of quitting smoking [44]. The effect of alcohol consumption on BMD is related to the amount of alcohol consumed, with moderate alcohol consumption slowing bone loss, while chronic excessive alcohol consumption may damage bone microstructure and increase fracture risk [45, 46]. An animal study showed that even without altering BMD, alcohol consumption altered the morphology and percentage of collagen in the trabeculae of the femoral neck in rats, increasing bone fragility [47].

## Conclusions

In conclusion, our study found that GNRI correlated with femoral neck BMD, and lumbar spine BMD, and did not correlate with total hip BMD after controlling for age as a confounding factor. Lower GNRI was associated with the development of osteoporosis in elderly type 2 diabetic patients. GNRI combined with age and albumin had better predictive ability for osteoporosis than PNI and CONUT scores. GNRI is more suitable for nutritional assessment in elderly type 2 diabetic patients.

## Limitations

There are some limitations of this study. First, it was a single-center retrospective study with an unbalanced gender distribution, which may have some bias; second, it could not prove a causal relationship between GNRI and osteoporosis in elderly patients with T2DM. Third, factors such as dietary habits, exercise intensity, and length of time receiving sunlight, which may affect bone density, were not considered.

## Data Availability

The datasets used/or analyzed during the current study are available from the corresponding author upon reasonable request.

## Abbreviations

TP: Total serum protein
ALB: Serum albumin
Cr: Creatinine
UA: Hematuria
FBG: Fasting blood glucose
TG: Triglycerides
TC: Total cholesterol
HDL: High-density lipoprotein
LDL: Low-density lipoprotein
BMI: Body mass index
GNRI: Geriatric Nutritional Risk Index
CONUT: Controlling nutritional status
PNI: Prognostic Nutrition Index
